# Therapeutic targeting of protein arginine methyltransferases reduces breast cancer progression by disrupting angiogenic pathways

**DOI:** 10.1016/j.bbrep.2025.102172

**Published:** 2025-07-31

**Authors:** Kamohelo Maphalala, Dakalo Portia Ramali, Lorraine Tshegofatso Maebele, Thanyani Victor Mulaudzi, Peace Mabeta, Zodwa Dlamini, Botle Precious Damane

**Affiliations:** a Department of Surgery, Steve Biko Academic Hospital, University of Pretoria, Hatfield, 0028, South Africa; b Angiogenesis Laboratory, Department of Physiology, Faculty of Health Sciences, University of Pretoria, Gezina, 0031, South Africa; c SAMRC Precision Oncology Research Unit (PORU), DSTI/NRF SARChI Chair in Precision Oncology and Cancer Prevention (POCP), Pan African Cancer Research Institute (PACRI), University of Pretoria, Hatfield, 0028, South Africa

**Keywords:** Breast cancer, Angiogenesis, Angiogenic signaling pathways, Hypoxia, Vascularization, Protein arginine methyltransferase inhibitors

## Abstract

Protein arginine methylation is an epigenetic modification involved in transcription, splicing and signal transduction and is mediated by protein arginine methyltransferases (PRMTs). PRMTs regulate various tumor angiogenesis pathways, including vascular endothelial growth factor receptor-2 (VEGFR-2) signaling. PRMT1, PRMT4, and PRMT5 activate distinct stages of angiogenesis. For example, inhibiting PRMT5 suppresses VEGF-induced vessel sprouting in experimental models while impairing hypoxia-inducible factor 1-alpha (HIF-1α) stability and VEGFR-2 phosphorylation. PRMT1 and PRMT4 similarly influence VEGF isoform expression, leading to increased angiogenesis. Targeting PRMTs in experimental models results in suppressed angiogenesis and reduced cancer progression. Several small-molecule PRMT inhibitors, including GSK3326595 and EPZ015666, have entered early-phase clinical trials for solid tumors. These agents show promise in inhibiting tumor angiogenesis, although there are toxicity concerns. This review examines the mechanistic basis and therapeutic rationale for targeting PRMTs in breast cancer and discusses combination approaches to overcome resistance. We integrate preclinical and emerging clinical data to highlight the potential antiangiogenic and tumor-suppressive effects of PRMT inhibitors, providing insights for future therapeutic strategies for breast cancer.

## Highlights

• Dysregulated arginine methylation may drive aberrant angiogenic signaling pathways in breast cancer.
• Arginine methylation controls endothelial cell functions via cytokines, growth factors, and related mediators.
• Inhibiting arginine methylation may help counteract dysregulated angiogenic signaling in breast cancer.

## 1 Introduction

Various angiogenic factors collectively stimulate endothelial cell proliferation, thereby increasing vascularity. Angiogenesis fuels tumor growth and progression, including breast cancer, by facilitating the formation of new blood vessels to meet the metabolic demands of proliferating cancer cells [1,2]. Breast cancer cells exploit angiogenic signaling pathways to accelerate growth and potentially invade nearby tissues. Like all tissues, breast cancer cells rely on the capillary network for sustained nutrition and oxygenation [3]. Initially, tumors do not promote angiogenesis and remain limited to a diameter of 1–2 mm due to inadequate oxygen and nutrient supply [4,5]. Balanced rates of cell proliferation and death characterize this dormant phase. Hypoxia in the tumor microenvironment (TME) restricts nutrient availability in the TME. The growing metabolic demands of tumor cells prompt an angiogenic switch, leading to sustained vascularization [4]. This switch stimulates the formation of new blood vessels, restoring nutrient and oxygen supply and accelerating tumor growth [6]. Key angiogenic growth factors, such as fibroblast growth factors (FGF) and vascular endothelial growth factor (VEGF), are pivotal in initiating and driving tumor angiogenesis [4,7]. Tumor cells synthesize pro-angiogenic factors that influence the existing vasculature and initiate this process. The balance between pro- and anti-angiogenic factors in the TME orchestrates the formation and stabilization of new blood vessels [8]. The critical role of angiogenic activators in malignant growth has been extensively studied [9].

Understanding the mechanisms underlying angiogenic signaling in breast cancer is vital for identifying novel therapeutic targets [10]. Tumor cells upregulate pro-angiogenic factors, such as VEGF and FGF, maintaining a heightened state of active angiogenesis [11]. Therapies targeting pro-angiogenic proteins have been developed [12] and are used to treat various advanced solid malignancies. These typically include monoclonal antibodies or tyrosine kinase inhibitors [10]. Anti-angiogenic drugs targeting angiogenic factors include bevacizumab, vatalanib, transforming growth factor-beta (TGF-β) antibodies, placental growth factor (PIGF) antibodies, trebananib, and human pattern recognition receptor long-pentraxin 3 (PTX3) [13]. Bevacizumab, also known as Avastin, was approved in 2008 by the United States Food and Drug Administration under the accelerated approval program for metastatic breast cancer. This approval was revoked in November 2011 due to safety and efficacy concerns [14]. Furthermore, the clinical use of these drugs is limited by their toxicity, adverse effects, acquired drug resistance, lack of predictive biomarkers [15], and low overall survival benefit [16]. Developing novel anti-angiogenic drugs with greater efficacy and reduced toxicity is vital for breast cancer patients [17,18].

Research on the inhibition of angiogenesis has expanded beyond targeting proteins and now includes epigenetic modifications. Epigenetic modifications are heritable changes in gene expression, which do not alter the primary DNA sequence [12]. Mechanisms controlling angiogenesis are tightly regulated at genetic and epigenetic levels [19]. Protein arginine methylation is an epigenetic modification mainly involved in transcriptional regulation, pre-mRNA splicing, and DNA damage response. This process is catalyzed by protein arginine methyltransferases (PRMTs), which transfer a methyl group from S-adenosylmethionine to the guanidino group of arginine residues on substrate proteins [20]. Various PRMTs regulate angiogenic signaling [21], and their expression and catalytic activity are frequently dysregulated in breast cancer [22]. Consequently, they have garnered attention as therapeutic targets for breast cancer. This review explores the mechanisms and regulation of protein arginine methylation, emphasizing its role in modulating angiogenic signaling pathways and promoting tumor progression. Furthermore, we evaluate the therapeutic potential of targeting PRMTs in cancer angiogenesis and highlight the associated challenges and limitations. By addressing these complexities, we aim to provide insights to inform future research directions and develop more effective therapeutic strategies.

## 2 Mechanisms and regulation of protein arginine methylation

### 2.1 Protein arginine methyltransferases, demethylases, and their substrates

Protein arginine methylation is a ubiquitous post-translational modification found in eukaryotes. PRMTs catalyze this modification, which involves the transfer of a methyl group from S-adenosyl-l-methionine (SAM) to the guanidino group of an arginine residue, generating an S-adenosyl-*l*-homocysteine and methylarginine [20,[23], [24], [25]]. Arginine methylation structurally modifies arginine residues by removing a potential hydrogen bond donor from the guanidino group without changing its net positive charge [23,25]. Mammalian cells contain nine PRMTs, which are classified into three types according to which arginine methylation they catalyze. Type I PRMTs (PRMT1, PRMT2, PRMT3, PRMT4, PRMT6, and PRMT8) catalyze the addition of a methyl group to either terminal guanidino nitrogen, generating a monomethyl arginine (MMA) and adding a second methyl group to the same guanidino nitrogen, generating asymmetric dimethylarginine (ADMA). Type II PRMTs (PRMT5 and PRMT9) catalyze the formation of MMA and the addition of a methyl group to the other terminal guanidino nitrogen to generate symmetric dimethylarginine (SDMA). The last category, Type III, includes only PRMT7 and catalyzes the formation of MMA [20,[23], [24], [25]].

PRMTs have various substrates involved in transcription, RNA metabolism, DNA repair, signal transduction, and cell cycle progression [26]. PRMTs mainly target proteins in the nucleus and cytoplasm containing glycine and arginine-rich (GAR) motifs. PRMT1, which has broad substrate specificity with over 40 targets, most being RNA processing proteins and multiple interacting partners, is responsible for over 85 % of arginine methylation. PRMT1, together with PRMT3 and PRMT6, methylates arginine-glycine-glycine repeats within GAR motifs. PRMT4 has a more restricted substrate preference, methylating arginine residues within proline-glycine-methionine-rich regions, primarily found in splicing factors. PRMT5 can methylate substrates containing GAR and non-GAR motifs, including proline-glycine-methionine regions. PRMT7 targets substrates consisting of pairs of arginine residues separated by one basic residue [27,28].

Protein arginine methylation can be reversed by demethylases, which remove methyl groups from arginine residues. Jumonji C domain-containing (JMJD) proteins represent one class of demethylases with multiple enzymatic functions, including arginine demethylase, lysine hydroxylase, tyrosine kinase activity, and protein clipping. JMJD6, for instance, demethylates non-coding RNA, histones, and non-histone proteins by directly converting methylarginine to arginine. JMJD1B is another JMJD protein with arginine demethylase activity [29,30]. Additionally, peptidyl arginine deaminase 4 is a histone demethylase that converts monomethylated arginine to citrulline [30]. PRMT4 and PRMT5 regulate gene expression through methylation of histones and splicing factors involved in transcriptional control. Their dysregulation is linked to tumor growth and faulty angiogenic signaling, making them promising epigenetic targets in breast cancer [31,32].

### 2.2 Regulatory processes

#### 2.2.1 Alternative splicing of PRMT isoforms

Alternative splicing of PRMT transcripts produces multiple isoforms with distinct structural features, enzymatic activities, and subcellular localizations [33]. PRMT1 has at least seven alternatively spliced variants, referred to as PRMT1-v1 through PRMT1-v7. These variants are generated by alternative splicing at the N-terminal region. These variants have different substrate specificities and subcellular distributions. For example, PRMT1-v1 and PRMT1-v2 are catalytically active, while PRMT1-v7 is not enzymatically active and may act as a dominant negative regulator [34,35]. PRMT1-v2 is primarily located in the cytoplasm due to the presence of a nuclear export sequence, whereas the other variants are predominantly nuclear [33].

Similar splicing variation has been reported for PRMT2, PRMT4 (coactivator-associated arginine methyltransferase 1 (CARM1), PRMT5, and PRMT7. These variants have different functions, with differences in their methyltransferase activity and interactions with target proteins [36,37]. For example, PRMT4 splice variants differ in their ability to methylate splicing factors and histones, thereby influencing transcription and RNA processing [31]. Isoform variation in PRMTs introduces functional diversity by changing subcellular localization, substrate specificity, and enzymatic activity [35,36]. These differences may influence how specific PRMT variants contribute to oncogenic processes, including regulating gene expression and RNA splicing. Such complexity poses a challenge for therapeutic targeting, as effective inhibitors may need to discriminate between functionally distinct isoforms to minimize off-target effects [38,39].

#### 2.2.2 Post-translational modifications

Phosphorylation of PRMTs can enable, inhibit, or switch their methyltransferase activity, depending on the modification site. PRMT1 and PRMT4 undergo phosphorylation as part of their regulatory mechanisms. Casein kinase 1 isoform alpha 1 phosphorylates PRMT1 at multiple sites, including regions 55–57, 102–105, and 284–289, influencing PRMT1's targeting to chromatin. Additionally, phosphorylation of PRMT1 at Y291 changes its substrate specificity.

PRMT4 is phosphorylated at residues S217 and S229, inhibiting its methyltransferase activity by promoting cytoplasmic localization or preventing dimerization. Furthermore, phosphorylation of PRMT4 at S572 by p38γ mitogen-activated protein kinase (MAPK) inhibits its translocation to the nucleus [33].

PRMT5 is phosphorylated at Y324 by proto-oncogene tyrosine-protein kinase (Src), which impedes its methyltransferase activity [39]. PRMT5 is also phosphorylated by liver kinase B1, suppressing its pro-tumoral enzymatic activity in breast cancer cells [40]. PRMT5 undergoes polyubiquitination and proteasomal degradation mediated by E3 ubiquitin ligase, namely the carboxyl terminus of heat shock cognate 70-interacting protein (CHIP) and the associated chaperone system [41]. CHIP also catalyzes the ubiquitination of PRMT, promoting its degradation [42].

Certain PRMTs undergo self-methylation to regulate their functions. Examples include, PRMT4, 6, and PRMT8. PRMT4 self-methylates at R551 within its C-terminal domain, a modification that does not affect catalytic activity but enhances protein-protein interactions necessary for transcriptional regulation. PRMT6 self-methylates at R35, which is critical for maintaining stability and, in disease contexts, for inhibiting the replication of human immunodeficiency virus-1. PRMT8 self-methylates within its N-terminal domain, a modification that blocks its catalytic site and prevents further methylation. PRMTs also methylate one another. For example, PRMT5 is asymmetrically methylated by PRMT4 at R505, promoting oligomerization and supporting its enzymatic function [33].

### 2.3 Mechanisms underlying protein arginine methylation dysregulation in cancer

#### 2.3.1 Aberrant PRMT expression

The expression of PRMT1-9 is commonly elevated in tumor tissues, and this upregulation is frequently linked to poor prognostic outcomes [43]. Numerous PRMTs are abnormally expressed in cancer cells, leading to the generation of cancer stem cells, epithelial-to-mesenchymal transition, and the proliferation of tumor cells. Reduced PRMT expression suppresses DNA repair genes and heightens cellular sensitivity to DNA-damaging agents [44].

Zhao et al. demonstrated that PRMT1 expression was significantly elevated in esophageal squamous cell carcinoma tissue samples, correlating with unfavorable clinicopathological features, including higher histologic grade, advanced tumor node metastasis stage, and poorer prognosis [45]. Similarly, PRMT1 is frequently overexpressed in other tumor types, including breast cancer, where its dysregulation contributes to oncogenic signaling pathways and cellular transformation [46]. In breast cancer, PRMT1 isoforms, particularly PRMT1-v1, are differentially expressed; however, the functional consequences and prognostic value of specific variants have yet to be fully characterized [36].

Although some transcriptomic studies suggest different isoforms have different functions and subcellular localization, their direct clinical significance requires further investigation [36,37]. Moreover, a certain level of arginine methylation is required to maintain cellular health. In mice, complete knockout of major PRMTs leads to mortality during embryonic development or shortly after birth, underscoring their important role in cellular viability and vascular development [46,48]. PRMTs are essential for cellular processes such as transcriptional regulation, RNA splicing, and DNA repair, and their complete inhibition is detrimental to normal cells. Consequently, PRMT-targeted therapies must be highly specific to minimize adverse effects on healthy tissues [47]. Selective inhibition of PRMT5 using small molecules such as EPZ015666 has demonstrated promising antitumor effects in preclinical models, including suppression of tumor growth and impaired cancer cell proliferation. Moreover, PRMT5 overexpression has been shown to promote angiogenesis via activation of the hypoxia-inducible factor 1-alpha (HIF-1α)/VEGF receptor (VEGFR)/protein kinase B (Akt) signaling pathway, emphasizing its role in tumor vascular remodeling and progression [48]. These findings underscore the therapeutic potential of targeting PRMT5 to disrupt aberrant angiogenesis in cancer.

#### 2.3.2 Mutations in PRMTs

Mutations in PRMT genes are less common than overexpression but can contribute to dysregulated methyltransferase activity in cancer. Several cancer-associated mutations have been identified in PRMT1 and PRMT5, particularly within or near the SAM binding site and dimerization domains, which are critical for enzymatic function.

Price et al. reported naturally occurring mutations in PRMT1-v2, including W215L, Y220 N, and M224V, which impair dimerization, reduce SAM affinity, and diminish methyltransferase activity [46]. Similarly, Rasheed et al. identified several important driver mutations in PRMT5 (D306H, L315P, and N318K) that affect its catalytic core. Computational protein modeling showed that these mutations may destabilize the active site, impair substrate methylation, or alter the binding of small-molecule inhibitors [49].

Although these mutations may change PRMT function [46,49], no experimental evidence currently connects them to angiogenesis-related pathways such as HIF-1α stabilization or VEGF/VEGFR signaling in breast cancer. Most research connecting PRMTs to angiogenesis focuses on overexpression or catalytic inhibition, particularly of PRMT5, rather than genetic mutations [21,50].

**Fig. 1:**
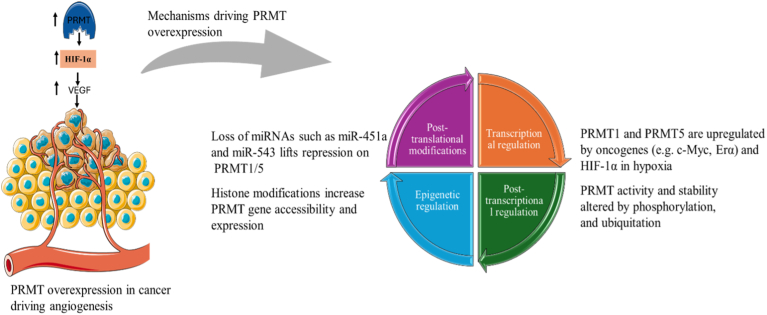
Protein arginine methyltransferase (PRMT)-specific regulatory mechanisms and their link to angiogenesis-related gene expression in cancer. PRMT expression and activity are modulated in cancer through transcriptional, epigenetic, post-transcriptional, and post-translational processes. These regulatory layers contribute to PRMT overexpression, which influences the transcription of angiogenesis-associated genes such as vascular endothelial growth factor A (VEGF-A) and hypoxia-inducible factor 1-alpha (HIF-1α). The diagram integrates upstream regulatory inputs with downstream transcriptional consequences, highlighting the contribution of PRMTs to tumor-associated angiogenic signaling.

The role of PRMT mutations in modulating angiogenic signaling remains an underexplored area in cancer research. Studies using site-directed mutagenesis, structural analysis, and activity assays demonstrate how single-point mutations can disrupt PRMT dimerization and enzymatic function, ultimately affecting downstream methylation targets [46]. Whether these mutations contribute to cancer progression or therapeutic resistance remains unanswered [47]. PRMT dysregulation in cancer is primarily driven by non-mutational mechanisms, including transcriptional activation, epigenetic remodeling, post-transcriptional derepression, and post-translational modifications [50]. These regulatory processes increase PRMT expression and activity in tumor cells and influence gene expression in angiogenic signaling [50,51] (Fig. 1).

## 3 Impact of protein arginine methylation on endothelial cell proliferation, migration, and tube formation

Tumor development involves genetic and epigenetic changes that promote oncogenes or suppress tumor suppressor genes. As a result, tumor cells proliferate and apoptosis decreases, leading to early hyperplastic growth. When the tumor reaches a critical size, cells distant from blood vessels lack adequate oxygen and nutrients, causing apoptosis or necrosis, which blocks further growth [52]. A tumor with limited nutrient and oxygen supply remains dormant, as the rate of cell death balances proliferation [53]. Nonetheless, tumor cells can circumvent this growth limitation through an angiogenic switch, a pro-angiogenic signaling state that promotes neovascularization. The newly formed vasculature restores oxygen and nutrient supply, freeing the tumor from dormancy and enabling rapid tumor growth [1,52]. Endothelial cell functions central to angiogenesis include proliferation, migration, and tube formation [54]. The balance between protein arginine methylation and demethylation is critical for regulating endothelial cell functions and maintaining vascular homeostasis [55].

### 3.1 Role of PRMTs in endothelial cell proliferation

Endothelial cell proliferation is an integral part of vascular growth and repair. Endothelial cells proliferate in response to various stimuli, such as tissue damage, inflammation, or hypoxia. These cells typically remain in a dormant G0 phase and re-enter the cell cycle upon the loss of cell-cell contact. Inhibiting the proliferation of endothelial cells is therefore a promising strategy for targeting tumor-induced angiogenesis [56,57,59]. TGF-β, a key angiogenic cytokine, can induce G1 cell cycle arrest in endothelial cells through activin receptor-like kinase 5 (ALK5)-mediated upregulation of cyclin-dependent kinase (CDK) inhibitors such as p15 and p21, particularly at high concentrations or during late-stage angiogenesis [57]. Activation of the TGF-β signaling pathway promotes immunosuppression, tumor invasion, metastasis, and angiogenesis [58]. TGF-β is overexpressed in breast cancer tissues compared to healthy breast tissue. Increased expression is associated with higher histological grade, axillary lymph node metastasis [59], and worse survival outcomes [59,60].

Several studies have reported the regulation of TGF-β by PRMTs. For example, Wei et al. reported that PRMT1 expression in hepatic cellular carcinoma cells correlated with TGF-β1 levels [61]. Liu et al. further demonstrated that PRMT5-mediated symmetric dimethylation of SMAD4 at arginine 361 enhanced TGF-β signaling activity in colorectal cancer [58]. The tumor suppressor protein p53 contributes to maintaining homeostasis [62]. p53 inhibits angiogenesis by interfering with the key hypoxia modulators, blocking the secretion of pro-angiogenic factors, and enhancing production of endogenous angiogenesis inhibitors [63]. The p53 and p21 signaling pathways are involved in triggering endothelial cell senescence. Experimental evidence shows that inhibiting p53 prevents senescence in endothelial cells exposed to stress stimuli [62].

Multiple studies have demonstrated that PRMTs regulate cell proliferation by modulating p53. For example, PRMT1 knockdown activates p53 signaling targets, leading to senescence and growth arrest. Mechanistically, PRMT1 binds to p53 and inhibits transcription, reducing the expression of downstream p53 targets [64]. In addition to PRMT1, PRMT6 also regulates P53 transcription. PRMT6 methylates histone H3 at arginine 2 on the p53 promoter, suppressing p53 transcription, suggesting that elevated PRMT6 levels may inhibit cell cycle arrest [65].

**Fig. 2:**
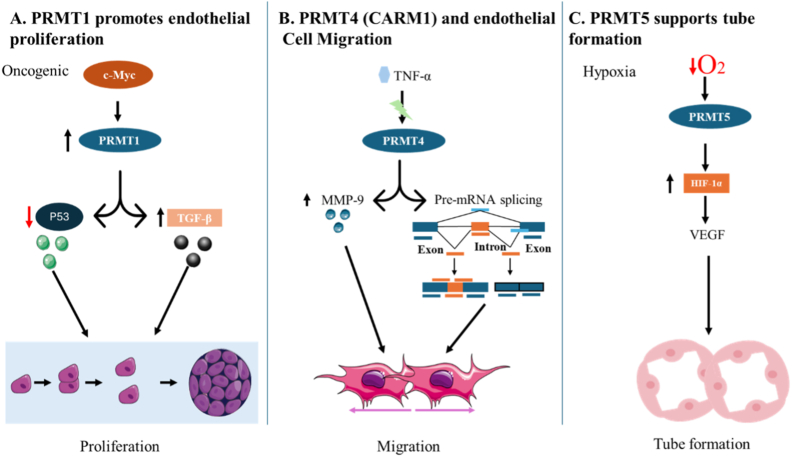
Protein arginine methyltransferase (PRMT)1, PRMT4, and PRMT5 regulate distinct stages of endothelial activation relevant to angiogenesis. This illustration shows the effects of different isoforms, PRMT1, PRMT4, and PRMT5, on endothelial cell behavior. PRMT1, transcriptionally induced by the oncogene c-MYC, promotes endothelial proliferation through transforming growth factor-beta (TGF-β) upregulation and p53 suppression. PRMT4, activated by inflammatory cytokines such as tumor necrosis factor-alpha (TNF-α), enhances endothelial migration by increasing matrix metalloproteinase-9 (MMP-9) expression and alternative splicing. PRMT5, upregulated under hypoxic conditions, facilitates tube formation by stabilizing hypoxia-inducible factor 1-alpha (HIF-1α) and increasing vascular endothelial growth factor (VEGF) expression.

PRMT5 has been shown to positively regulate p53 expression, translation, and function. PRMT5 knockdown induces G1 arrest and inhibits cell proliferation [66,68]. The isoform-specific contributions of PRMT1, PRMT4, and PRMT5 to endothelial cell proliferation, migration, and tube formation are illustrated in Fig. 2. Other pathways that regulate endothelial cell proliferation, including VEGF [67], Wnt/β-catenin, and notch signaling [45], are also regulated by PRMTs. Targeting PRMT1 and PRMT5 may restore p53 function [68] and attenuate TGF-β-mediated angiogenic responses, offering a rational therapeutic strategy in breast cancer [20]. Furthermore, pharmacological inhibition of PRMT5 in vitro reportedly reduces endothelial cell proliferation and VEGF-induced aortic sprouting, supporting its role as a viable anti-angiogenic target [21].

### 3.2 PRMTs induce endothelial cell migration and tube formation

Endothelial cell migration refers to the movement of endothelial cells lining the blood vessels from one location to another. This process is essential for angiogenesis, wound healing, and inflammation and is often initiated by stimuli such as growth factors, e.g., VEGF, chemokines, or mechanical cues. Migration relies on dynamic cytoskeletal rearrangements that extend and retract cell protrusions, interactions with cell-matrix adhesion molecules such as integrins, and the coordinated activity of cell-cell junctions that support collective movement as sheets or cords [69,70]. Proteolytic enzymes secreted by endothelial cells degrade the extracellular matrix (ECM), clearing pathways through tissue. Tube formation, a key step in angiogenesis, involves assembling endothelial cells into hollow tubular structures that form the framework of new blood vessels. This process depends on cell–cell and cell–matrix adhesion molecules (e.g., via integrins), cytoskeletal remodeling, and the reordering of ECM components to create a central lumen [55].

During angiogenesis, matrix metalloproteinases (MMPs), particularly MMP-9, are critical for basement membrane degradation, ECM remodeling, detachment of pericytes, release of matrix-bound growth factors, and the cleavage of endothelial junctions, all of which facilitate migration and tube formation [71]. MMP-9 is regulated by PRMT7 [72] and PRMT6, the latter also controlling MMP-2 expression [73] and thrombospondin-1, a potent endogenous angiogenesis inhibitor [51]. The phosphoinositide 3-kinase (PI3K)/Akt/mammalian target of rapamycin (mTOR) pathway further supports endothelial migration [74] and tube formation [75]. Tan et al. demonstrated that PRMT5 knockdown reduced PI3K levels and Akt/mTOR phosphorylation in bladder cancer cells, while PRMT5 had the opposite effect [76].

## 4 Regulation of angiogenic signaling pathways by PRMTs

### 4.1 VEGF/VEGFR

VEGF-A was the first identified member of the VEGF family, which also includes VEGF-B, VEGF-C, VEGF-D, VEGF-E (viral VEGF), VEGF-F (snake venom VEGF), placental growth factor, and endocrine gland-derived VEGF. VEGF exerts its effects primarily through tyrosine kinase receptors VEGFR-1 and VEGFR-2, which are expressed on endothelial cells, neurons, hepatocytes, mast cells, hematopoietic stem cells, osteoblasts, and retinal pigment epithelium cells [77]. VEGF-C and VEGF-D are lymphangiogenic factors that bind to VEGFR-3, predominantly expressed on lymphatic endothelial cells, to stimulate lymphangiogenesis. VEGF-B and placental growth factor bind selectively to VEGFR-1 and are mainly involved in vascular remodeling rather than angiogenesis [10].

VEGF-A is secreted by endothelial cells and by thrombocytes, macrophages, dendrocytes, astrocytes, osteoblasts, lymphocytes, and tumor cells under hypoxic conditions [78]. VEGF-A is a potent angiogenic factor that drives tumor initiation, progression, and metastasis [79]. Overexpression of VEGF commonly occurs before the invasion of breast cancer cells and is associated with tumor size and poor prognosis [10]. VEGF-A is overexpressed in breast tumors compared to normal breast tissues [80]. VEGF-A acts as an autocrine for tumor cell survival and may influence tumor progression from carcinoma in situ to an invasive form [81]. In addition to promoting endothelial proliferation and migration, VEGF-A increases vascular permeability and activates macrophages and granulocytes. Tumor cells also produce factors that initiate neovascularization and inhibit endogenous angiogenesis suppressors [78].

Full-length VEGFR-2 comprises extracellular, transmembrane, juxtamembrane, catalytic tyrosine kinase domains, and a flexible C-terminal region [82]. Ligand binding induces receptor dimerization and *trans*-autophosphorylation of tyrosine residues. Of the 19 tyrosine residues in VEGFR-2, five major phosphorylation sites (Y951, Y1054, Y1059, Y1175, and Y1214) regulate signaling. Y1054 and Y1059, located in the activation loop, are critical for kinase activity. Phosphorylation of Y1175 in the carboxy-terminal domain generates binding sites for phospholipase C gamma (PLCγ), the p85 subunit of PI3K, SH2 domain-containing adaptor protein B (SHB), and Shc-like protein, and is a key mediator of VEGFR-2 signaling. Y1214 is required for binding the noncatalytic region of tyrosine kinase (NCK) [82,83]. Other important sites include Y801 (juxtamembrane domain), which activates PLCγ, and Y951 (kinase insert domain), which mediates signaling through T cell-specific adapter protein (TSAd) [82].

Upon recruitment to activated VEGFR-2, PLCγ becomes phosphorylated and hydrolyzes phosphatidylinositol-4,5-biphosphate into inositol 1,4,5-trisphosphate (IP3) and diacylglycerol (DAG). IP3 mobilizes calcium from the endoplasmic reticulum, increasing intracellular calcium, while DAG activates protein kinase C (PKC). PKC, in turn, stimulates the accelerated fibrosarcoma 1/mitogen-activated protein kinase/extracellular signal-regulated kinase 1/2 pathway, leading to the transcriptional regulation of genes involved in endothelial proliferation [83]. PKC activation also stimulates endothelial nitric oxide synthase (eNOS), increasing nitric oxide (NO) production and vascular permeability [84]. Additional pathways downstream of VEGFR include TSAd/Src/PI3K/Akt, SHB/focal adhesion kinase/paxillin, SHB/PI3K/Rac, and NCK/p38/MAPKPK2, all of which contribute to angiogenesis [11,82]. VEGF/VEGFR signaling depends on the coordinate interaction of these proteins [82], and disruption at any step can impair the pathway [85].

Several studies have implicated PRMTs in regulating VEGF signaling. Zhang et al. reported that PRMT3 overexpression enhanced VEGF signaling via stabilization of HIF-1α stability [86]. PRMT6 promotes alternative splicing of VEGF pre-mRNA, favoring exon 6 skipping and generating VEGF165 [87], an isoform associated with tumor progression and poor prognosis [88]. PRMT4 also contributes to VEG165 expression [87]. Increased VEGF was observed in mice with lung tumors overexpressing PRMT6 after tamoxifen and urethane treatment, consistent with enhanced angiogenesis [89]. Conversely, PRMT1 silencing in melanoma cells upregulated VEGF, suggesting context-dependent regulation [90].

PRMTs methylate VEGFR-2 at arginine residues (Arg817, Arg1115) and by lysine methyltransferases at lysine residues (Lys856, Lys861, and Lys1041). Methylation at Arg817 influences phosphorylation of Y820, promoting recruitment and activation of Src kinase. Activated Src regulates endothelial filopodia formation, sprouting, and tube assembly [55]. Collectively, these findings highlight the role of PRMTs in modulating VEGF/VEGFR-driven angiogenesis. Targeting PRMT-mediated methylation of VEGFR-2 may offer a strategy to disrupt abnormal VEGF signaling in cancer. Preclinical studies have shown that PRMT3 can methylate HIF-1α, preventing its degradation and increasing VEGF expression under hypoxia [91]. PRMT6 regulates VEGF splicing to produce VEGF165 [51], while PRMT1 and PRMT5 modulate VEGFR2 activity. PRMT5 promotes VEGFR-2 phosphorylation and downstream PI3K/Akt/eNOS activation, driving endothelial proliferation and vascular remodeling [92].

### 4.2 Fibroblast growth factors and receptors

The fibroblast growth factor and receptor (FGF/FGFR) signaling axis regulates various cellular processes, including angiogenesis, differentiation, embryonic development, proliferation, survival, and migration [93]. The human FGFR family comprises four highly conserved receptor tyrosine kinases, namely FGFR1, FGFR2, FGFR3, and FGFR4, encoded by different genes. FGFR5, which lacks an intracellular kinase domain, is a decoy receptor that binds to FGF ligands and may block their interaction with signaling-competent FGFRs. FGFR1–4 share a typical structure consisting of an extracellular domain, a transmembrane region, and a cytoplasmic tyrosine kinase domain [94]. The extracellular portion has three immunoglobulin (Ig)-like domains, IgI, IgII, and IgIII, with IgII and IgIII mediating FGF ligand binding. FGFs and their receptors are expressed in tissue-specific patterns, and this expression, along with differences in binding affinity and alternative splicing, determines the specificity of ligand–receptor interactions [95].

FGFs, particularly FGF2, were among the first pro-angiogenic factors identified and directly regulate tumor angiogenesis across all stages. Endothelial cells predominantly express FGFR1 rather than FGFR2, although activation of both receptors drives the development and maintenance of tumor vasculature [93]. FGFs are released from the ECM by heparins, proteases, or specific FGF-binding proteins. They then interact with FGFRs to form stable complexes with heparin sulfate proteoglycans on the cell surface. Klotho family proteins further enhance FGF–FGFR binding by associating with receptors and increasing ligand affinity [95]. Upon ligand binding, FGFRs dimerize and undergo phosphorylation of their intracellular tyrosine kinase domains. These phosphorylated domains recruit downstream signaling proteins, including FGFR substrate 2 and PLCγ, activating several transduction pathways, including RAS-MAPK, PI3K-AKT, IP3–Ca2+, DAG-PKC, and Janus kinase–signal transducer and activator of transcription (JAK-STAT) [94,95].

Several studies have demonstrated that PRMT5 regulates FGF/FGFR signaling. In lung cancer, PRMT5 expression was positively correlated with FGFR3 levels and associated with poor prognosis. Mechanistically, PRMT5 repressed the transcription of the miR-99 family through symmetric dimethylation of histone H4 at arginine 3, leading to increased FGFR3 expression and activation of the ERK1/2 and Akt pathways [96]. Similar correlations between PRMT5 and FGFR3 or PI3K/AKT/mTOR and ERK signaling have been reported in non-small cell lung cancer and colorectal cancer cell lines [97,98]. In addition to regulating FGFR3, PRMT5 has been shown to modulate the transcriptional activity of FGFR1, FGFR2, and FGFR4 [99,100]. These findings suggest that elevated PRMT5 expression enhances FGF/FGFR signaling, contributing to tumor angiogenesis and progression.

### 4.3 Hypoxia-inducible factors

HIFs are heterodimeric transcription factors composed of three α and β subunits: HIF-1α, HIF-2α, and HIF-3α, and their respective partners HIF-1β, HIF-2β, and HIF-3β. HIFs regulate oxygen homeostasis by controlling the expression of more than 1000 genes related to various key cellular functions, such as angiogenesis, metabolic remodeling, differentiation, and migration [101]. HIF target genes include VEGF, transferrin, transferrin receptors, erythropoietin, glycolytic enzymes, anti-apoptotic proteins, TGF-β, platelet-derived growth factor-B, insulin-like growth factor-2, and epidermal growth factor [102]. HIF-1α is expressed ubiquitously, whereas HIF-2α is mainly found in highly vascularized organs such as the brain, lung, liver, heart, intestines, kidney, pancreas, and uterus [101]. HIF-2α is also present in subsets of tumor-associated macrophages, while HIF-3α is expressed primarily in pulmonary alveolar epithelial cells and the kidney [103].

**Fig. 3:**
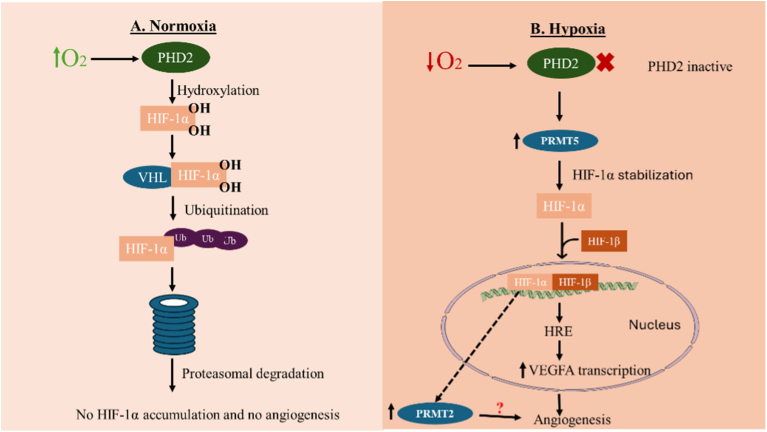
Regulation of hypoxia-inducible factor 1-alpha (HIF-1α) under normoxic and hypoxic conditions. Under normoxia, HIF-1α is hydroxylated on specific proline residues by prolyl hydroxylase domain protein 2 (PHD2), facilitating recognition by the von Hippel–Lindau E3 ubiquitin ligase complex. This modification triggers polyubiquitination and proteasomal degradation of HIF-1α, preventing its transcriptional activity. Under hypoxic conditions, PHD2 activity is suppressed, leading to HIF-1α stabilization, enabling nuclear accumulation, dimerization with HIF-1β, and transcriptional activation of target genes, including vascular endothelial growth factor A (VEGFA). HIF-1α has also been shown to upregulate PRMT2 in glioblastoma, although the relevance of this interaction in endothelial angiogenesis remains unclear.

HIF-1α is a potent pro-angiogenic factor characterized by aberrant accumulation under hypoxic conditions, where it promotes the transcription of numerous angiogenic and survival genes [86]. Tumor hypoxia contributes to therapy resistance, metastasis, heterogeneity, and overall progression, and is recognized as a marker of poor prognosis [104]. HIF-1α protein levels and activity are regulated at multiple stages. Under normoxia, HIF-1α is constitutively transcribed and synthesized but rapidly degraded, with a half-life of about 5 min. Degradation occurs via hydroxylation by prolyl hydroxylase domain protein 2 (PHD2), which targets HIF-1α for polyubiquitination by the Von Hippel-Lindau complex and subsequent proteasomal degradation. However, in low oxygen conditions, several pathways regulate the stability and transcriptional activity of HIF-1α through post-translational modifications [105]. Under hypoxia, PHD2 is inactive [86], allowing HIF-1α to escape degradation, translocate to the nucleus, dimerize with HIF-1β, and drive transcription of target genes [86,101] (Fig. 3). Although the α-β dimer functions as the primary transcriptional complex [101], individual subunits can also influence gene expression to maintain oxygen homeostasis [104].

HIF dysregulation is common in cancer and linked to increased aggressiveness, metastasis, therapy resistance, and poor outcomes. Understanding HIF regulation is therefore essential for developing targeted therapies [106]. Post-translational modifications are especially important for controlling HIF function and mediating the transition from HIF-1α to HIF-2α expression during chronic hypoxia [101]. In breast cancer, genetic changes frequently increase HIF-α stability and activity independently of oxygen availability. These include hyperactivation of the PI3K/Akt/mTOR or MAPK pathways and loss of tumor suppressors such as phosphatase and tensin homolog, p53, or breast cancer susceptibility gene 1. HER2 overexpression, found in 15–30 % of human breast cancers, and estrogen receptor-α, positive in 70 % of breast cancers, also increase HIF-α levels via increased PI3K/Akt/mTOR signaling. Notably, estrogen receptor-α selectively promotes HIF-1α expression through an estrogen response element within the HIF-1α promoter, without similarly affecting HIF-2α [16].

Several studies have shown that PRMTs regulate HIF-1α stability and function. For example, Liao et al. demonstrated that PRMT3 directly methylates HIF-1α at arginine 282, preventing polyubiquitination and promoting protein stabilization [107]. PRMT3 overexpression increased HIF-1α levels, while depletion or pharmacological inhibition decreased its expression in glioblastoma models [86]. Stabilized HIF-1α promotes angiogenic gene transcription and tumor adaptation. PRMT5 expression also increases during hypoxia and supports HIF-1α stabilization, thereby stimulating VEGF production and signaling. PRMT5 further regulates phosphorylation of VEGFR, Akt, and eNOS. Inhibition of PRMT5 pharmacologically or by shRNA reduced VEGFR2 phosphorylation at Tyr1175 and Tyr966, Akt phosphorylation at Thr308 and Ser473, and eNOS phosphorylation at Ser1177, highlighting its broad role in this pathway [92].

HIF-1α can also activate PRMT2 expression under hypoxic conditions [108] (Fig. 3). Beyond direct methylation, PRMTs modulate hypoxia-responsive transcription by changing chromatin accessibility. For example, PRMT5 methylates histone H3 and H4 arginine residues, increasing the expression of glycolytic and angiogenic genes [109]. PRMT2, though not directly methylating HIF-1α, functions as a nuclear receptor coactivator and contributes to chromatin looping, further enhancing hypoxia-driven transcription [109]. These findings demonstrate that PRMTs regulate HIF-1α stability, transcriptional activity, and downstream angiogenic signaling, thereby contributing to tumor progression and therapy resistance in breast cancer.

## 5 Therapeutic targeting of PRMTs in cancer angiogenesis

### 5.1 Targeting PRMTs impairs angiogenic pathways in cancer

The inhibition of angiogenesis suppresses the production and activity of pro-angiogenic factors secreted by tumor cells and blocks their receptors on endothelial cells. Consequently, angiogenesis inhibition deprives tumors of nutrients required for growth and promotes tumor vasculature to improve the delivery of therapeutic agents. Although anti-angiogenic therapies have shown efficacy in various solid tumors, they have not improved survival outcomes in breast cancer [10]. Highly potent and selective small-molecule PRMT inhibitors have been developed since the early 21st century [110]. Investigating PRMT function in vivo and in vitro, combined with these selective inhibitors, may help elucidate their distinct and overlapping pro-tumorigenic roles in breast cancer.

In pre-clinical studies, small-molecule inhibitors targeting PRMT3, PRMT4, PRMT5 and PRMT6 have demonstrated therapeutic potential [111]. PRMT5 inhibitors, in particular, reduce enzymatic activity as evidenced by decreased levels of symmetric dimethylarginine [92]. GSK3326595 is a potent, reversible, and selective PRMT5 inhibitor with pro-apoptotic and anti-proliferative activity in multiple solid tumor models [112]. This compound has advanced to clinical trials in early-stage breast cancer and other malignancies (NCT04676516). A phase I study in adults with advanced solid tumors, including breast cancer, reported manageable adverse events such as fatigue, anemia, nausea, alopecia and dysgeusia. Grade 3–4 events such as anemia, fatigue, thrombocytopenia and neutropenia were observed, though no treatment-related deaths occurred [113].

EPZ015666 (GSK3235025) is another selective PRMT5 inhibitor under clinical evaluation [21,92]. In preclinical models, EPZ015666 suppressed proliferation and tumor progression in triple-negative breast cancer cells [114]. It also inhibited VEGF-induced aortic ring sprouting and endothelial tube formation in a dose-dependent manner, while downregulating VEGF/PI3K/eNOS signaling. Additionally, PRMT5 inhibition reduced HIF-1α expression and stability [21,92], suppressing the VEGF/VEGFR pathway. GSK591, another PRMT5 inhibitor, similarly disrupts VEGF/VEGFR signaling [92]. Estrogen also plays a vital role in angiogenesis by suppressing the expression of soluble VEGFR-1, thereby promoting neovascularization [115]. PRMTs have been implicated in regulating estrogen signaling [[116], [117], [118]]. Le Romancer et al. demonstrated that PRMT1 methylates estrogen-α at arginine 260, promoting its cytoplasmic localization during rapid estrogen signaling [119]. This methylation was blocked by adenosine dialdehyde, a general methylation inhibitor, in MCF-7 breast cancer cells. Mei et al. further demonstrated that PRMT5 knockdown reduced estrogen-α expression at the mRNA and protein levels [120]. Thus, targeting PRMTs could indirectly inhibit angiogenesis by disrupting estrogen signaling in breast cancer.

**Fig. 4:**
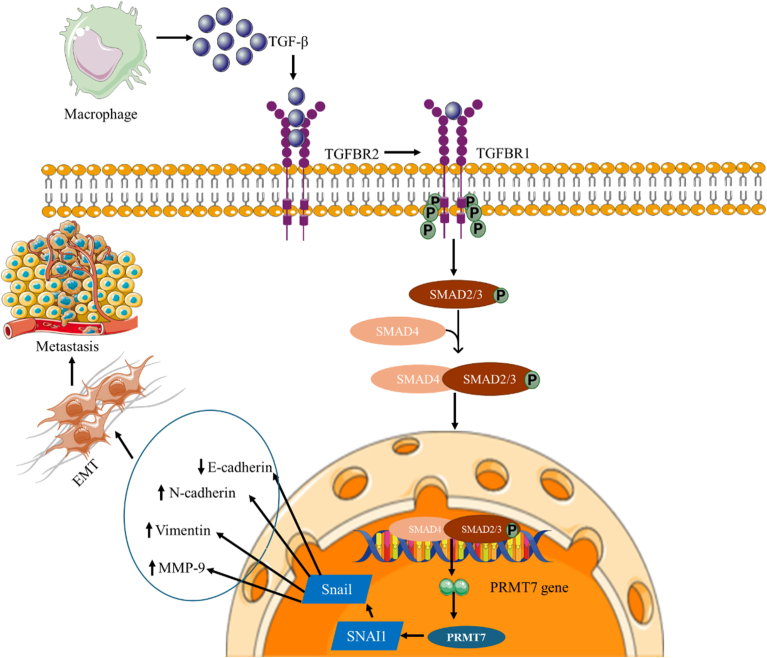
PRMT7-SMAD-SNAI1 signaling cascade promoting epithelial-to-mesenchymal transition and metastasis in breast cancer. PRMT7 facilitates the nuclear translocation of SMAD2, SMAD3, and SMAD4 following activation of the transforming growth factor-beta (TGF-β) receptor. This signaling promotes upregulation of snail family transcriptional repressor 1 (SNAI1), a transcription factor that represses E-cadherin and induces the expression of mesenchymal genes such as matrix metalloproteinase-9 (MMP9), N-cadherin, and vimentin. These changes drive epithelial–mesenchymal transition and metastatic dissemination in breast cancer.

Inhibiting arginine metabolism has also shown potential for reducing tumor angiogenesis. However, this approach is complex and sometimes controversial. PRMT1 catalyze arginine methylation to generate ADMA [121], a competitive inhibitor of eNOS. Arginine is an eNOS substrate, generating NO and l-citrulline [122,123]. NO promotes angiogenesis through several mechanisms: dilating arterioles to increase tumor perfusion, enhancing vascular permeability, stimulating VEGF secretion via the NO/cyclic guanosine monophosphate pathway, and activating cyclooxygenase-2 to produce additional pro-angiogenic factors [124]. Increased methylation elevates ADMA levels, thereby reducing NO production [121]. Consequently, PRMT inhibition could lower ADMA, relieve eNOS inhibition, and paradoxically increase NO-mediated angiogenesis [125]. Overall, these studies illustrate the therapeutic potential of PRMT inhibitors to counteract aberrant proangiogenic signaling and limit breast tumor progression (Fig. 4). However, few studies have comprehensively evaluated the effects of PRMT inhibition on angiogenesis in cancer. Additional research is needed to clarify whether targeting specific PRMTs consistently inhibits or promotes angiogenesis and to define their mechanistic roles in breast cancer using in vitro and in vivo models, ultimately informing the design of clinical trials.

Emerging preclinical and early-phase clinical studies support the therapeutic relevance of PRMTs in angiogenesis. For example, inhibiting PRMT5 using EPZ015666 significantly impairs VEGF-induced aortic sprouting and endothelial tube formation [21]. GSK3326595 has been shown to suppress HIF-1α and VEGF expression under hypoxic conditions, highlighting its potential as an anti-angiogenic therapy in breast and other solid tumors [113,126]. Ongoing trials are evaluating additional PRMT inhibitors, including GSK3368715 (targeting PRMT1) and CMP5 (targeting PRMT5), in patients with advanced malignancies, including breast cancer [[127], [128], [129]]. Beyond angiogenesis, certain PRMTs also regulate metastatic progression [36]. Notably, PRMT7 interacts with SMAD2, SMAD3, and SMAD4 following TGF-β receptor activation, enhancing their nuclear translocation and driving transcription of SNAI1. This activity suppresses E-cadherin while upregulating mesenchymal markers such as MMP9, facilitating epithelial-to-mesenchymal transition [130,131], a key mechanism of metastasis.

### 5.2 Combining PRMT inhibitors with other drugs to mitigate breast cancer

The broad effects of PRMT inhibition provide a strong rationale for incorporating these agents into combination therapies, which may enhance antitumor efficacy while reducing toxicity [47]. Several studies have demonstrated the benefits of combining PRMTs with other therapies, including chemotherapy, immunotherapy, and additional PRMT inhibitors, across various tumor models. For example, dual targeting of PRMT1 and PRMT5 has produced synergistic inhibition of tumor growth [128]. In pancreatic ductal adenocarcinoma, the combination of MS023 (a PRMT1 inhibitor) and EPZ015666 (a PRMT5 inhibitor) resulted in enhanced antitumor activity [132]. PRMT inhibitors have also been evaluated in combination with chemotherapeutic agents such as gemcitabine in pancreatic ductal adenocarcinoma [132], and cisplatin, doxorubicin, and camptothecin in breast cancer [133]. Notably, PRMT5 has been implicated in mediating doxorubicin resistance in breast cancer [134], suggesting that combining PRMT5 inhibitors with chemotherapy could synergistically suppress breast cancer cell proliferation and growth [135]. Collectively, these findings highlight the potential of combination strategies involving PRMT inhibitors to improve therapeutic outcomes in breast cancer and to target angiogenesis more effectively. Clinical trials evaluating several of these inhibitors, including GSK3326595 and EPZ015666, and their mechanistic links to angiogenic signaling in breast cancer are summarized in Table 1.

**Table 1:** Selected clinical trials evaluating protein arginine methyltransferase (PRMT) inhibitors with mechanistic relevance to angiogenesis in breast cancer.

Inhibitor	PRMT Target	Cancer type	Phase	Mechanism	Trial Number	References
**GSK3326595 (EPZ015938)**	PRMT5	Solid tumors and non-Hodgkin lymphoma; ongoing trials in breast cancer	II	Inhibiting PRMT5 reduces HIF-1α stability and VEGF expression under hypoxia, impairing tumor angiogenesis.	NCT04676516	[113,126]
**JNJ-64619178**	PRMT5	Solid tumors, breast cancer, adenoid cystic carcinoma, prostate cancer	II	In triple-negative breast cancer models, PRMT5 inhibition by JNJ-64619178 reduces KEAP1 methylation, enhancing ferroptosis and potentially impairing tumor angiogenesis.	NCT03573310	[136,137]
**GSK3368715**	PRMT1	Breast cancer, solid tumors	I	PRMT1 may regulate HIF-1α indirectly via TGF-β/p53 axes, affecting angiogenesis pathways.	NCT03666988	[127,128]
**CMP5**	PRMT5	Breast cancer, glioblastoma	II	PRMT5 inhibition decreases histone methylation at DKK1/3 promoters, reducing Cyclin D1 and SURVIVIN expression, which are involved in angiogenesis.	NCT05952557	[129]

## 6 Challenges and limitations

### 6.1 Scarcity of data on epigenetic regulation in the TME

**Fig. 5:**
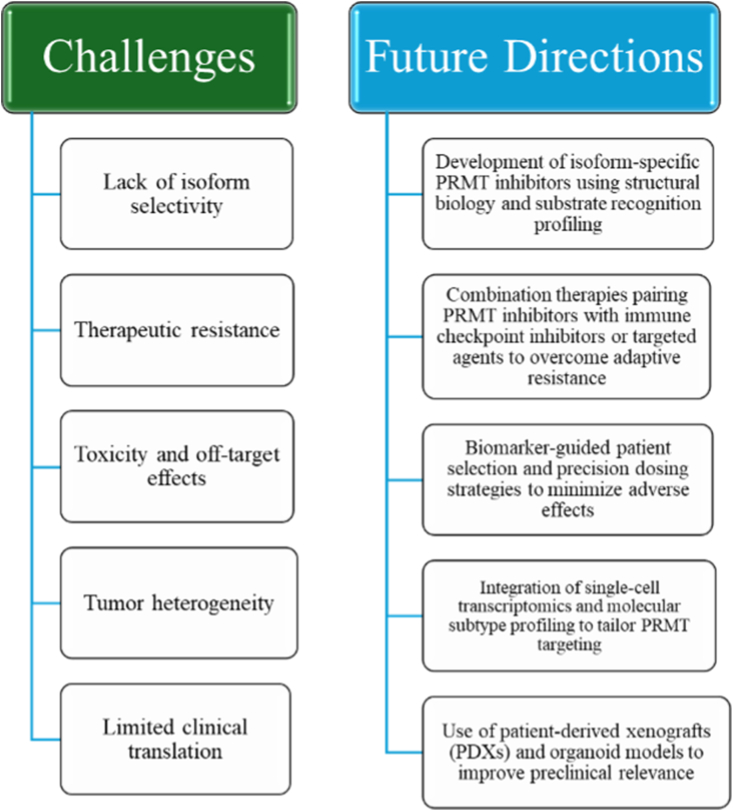
Current challenges and future directions for protein arginine methyltransferase (PRMT) inhibitor therapy in breast cancer. The main barriers to the clinical application of PRMT inhibitors are presented alongside strategies and opportunities to address them and enable their eventual translation into effective treatments.

The TME is a critical factor in tumorigenesis and cancer progression and must be considered in therapeutic development. The complexity of epigenetic regulation and its interplay with genetic alterations makes it challenging to delineate the fundamental relationships between epigenetic modifications and breast cancer development. Additionally, the dynamic nature of the environment, including diet, lifestyle, hormonal influences, and the gut microbiome, further shapes epigenetic changes and immune responses in breast cancer [138] (Fig. 5).

The interactions between PRMT activity and processes such as immune cell infiltration, stromal remodeling, and metabolic reprogramming within the TME remain poorly understood. A more detailed characterization of how specific PRMT isoforms function in distinct cellular components of the TME could inform the design of personalized therapeutic strategies in breast cancer [139].

### 6.2 Selectivity challenges leading to toxicity

Limited selectivity and excessive inhibition of PRMTs can lead to unintended effects on healthy cells, including dysregulated proliferation. PRMTs are essential for maintaining adult hematopoietic cell populations, so their broad inhibition is associated with hematologic toxicity, including anemia, thrombocytopenia, and neutropenia [139]. For example, a phase 1 trial of GSK3368715, a PRMT1 inhibitor in patients with advanced solid tumors was terminated due to a higher-than-expected incidence rate of thromboembolic events, insufficient target engagement in the TME at tolerable doses, and limited clinical efficacy [127].

Additionally, there is a lack of comprehensive pharmacodynamic and pharmacokinetic data on PRMT inhibitors in in vivo models. Developing next-generation inhibitors with improved selectivity and favorable pharmacologic profiles will be essential to enable low-dose administration and mitigate dose-limiting toxicities. Emerging strategies to enhance specificity among PRMT isoforms and between malignant and healthy cells include allosteric inhibition, dual-target inhibition, covalent inhibition, protein degradation, and PRMT5-linker PPI inhibition [140].

### 6.3 Limited studies on PRMT function

The functional mechanisms of PRMTs remain incompletely characterized. For example, the distinct roles of nuclear versus cytoplasmic PRMTs are not fully understood [129]. Many PRMTs share conserved sequence motifs with high homology, resulting in functional cross-talk. Inhibiting one PRMT isoform can inadvertently affect the activity of others [32,44]. However, the precise mechanisms underlying this crosstalk are poorly defined, which poses challenges for developing truly selective inhibitors [32]. Although thousands of arginine methylation substrates and sites have been identified, much work remains to elucidate the biological relevance of individual methylation events. Not all arginine methylation marks may be functionally significant, and some may represent biochemical noise. Discriminating between essential and nonessential arginine methylation sites remains a major challenge.

Current research has primarily focused on the enzymatic roles of PRMTs, but their potential non-enzymatic functions warrant further investigation. For instance, PRMT8 possess phospholipase activity via an HKD characteristic of phospholipase D enzymes, an activity unique among PRMTs [141]. Additionally, PRMT1 exists in multiple isoforms, some exhibiting low enzymatic activity, and their functions remain unidentified [34,141]. Future studies are needed to advance understanding of PRMTs in both normal cellular processes and disease, offering broader insights into their contributions to cancer pathogenesis and therapy.

## 7 Future directions

Despite advances in understanding the roles of PRMTs in cancer, numerous biological and translational challenges must be addressed to fully harness these enzymes as therapeutic targets in angiogenesis-driven breast tumors. One of the most pressing issues is the lack of isoform-specific PRMT inhibitors. Most current compounds target the conserved catalytic methyltransferase domain shared across all PRMTs. Several PRMTs, including PRMT1, PRMT4, and PRMT5, exist in various isoforms generated by alternative splicing, each with potentially distinct functions, substrate specificity, and cellular localization [35,36]. The roles of these isoforms in angiogenesis and therapy response remain poorly characterized. Future research should prioritize defining isoform-specific expression patterns across breast cancer subtypes and under hypoxic conditions, using transcriptomic and proteomic profiling. Such insights are essential to inform the rational design of isoform-selective therapeutics.

Additionally, functional crosstalk between PRMTs complicates efforts to develop selective interventions. PRMTs often operate in overlapping pathways, targeting shared histone and non-histone substrates [46,126]. For example, PRMT1 and PRMT6 regulate chromatin structure through methylation of H4R3, while PRMT4 and PRMT5 modulate transcriptional coactivator complexes. Preclinical studies have shown that dual inhibition of PRMT1 and PRMT5 can produce synergistic tumor suppression [122,123], but the broader consequences of disrupting such interactions on normal tissue homeostasis require further investigation.

Tumor heterogeneity is a barrier to effective PRMT-targeted therapy. PRMT expression varies across breast cancer subtypes (e.g., luminal, HER2-enriched, triple-negative) and across spatial domains within the same tumor [133]. Moreover, the TME, including stromal fibroblasts, endothelial cells, and immune infiltrates, can modulate PRMT activity and expression in response to hypoxia, cytokines, and metabolic stress. Addressing these context-dependent effects will require high-resolution approaches such as single-cell transcriptomics, methylation mapping, and spatial epigenomics to identify vulnerabilities that are both subtype-specific and microenvironment-informed [53].

From a therapeutic perspective, there is a critical need for next-generation PRMT inhibitors with improved pharmacokinetics, reduced hematologic toxicity, and enhanced tumor penetration (Fig. 5) [47]. While several compounds have shown promising preclinical efficacy, many have failed to advance to trials due to dose-limiting toxicities or insufficient efficacy in solid tumors. Innovative strategies, such as allosteric inhibitors, substrate-competitive inhibitors, and targeted protein degraders (e.g., PROTACs), are currently under investigation [135]. Combining PRMT inhibitors with other therapies, including chemotherapy, anti-angiogenic agents, and immunotherapies, may improve clinical outcomes and help overcome resistance [113,126]. Ultimately, translating PRMT inhibitors from promising targets into effective clinical tools will require a deeper understanding of their biology in heterogeneous tumor systems, as well as the integration of molecular profiling into therapeutic design and patient selection.

## 8 Conclusion

PRMTs play an important role in regulating angiogenesis and tumor progression in breast cancer. Despite the therapeutic potential of PRMT inhibitors, challenges such as isoform specificity, functional redundancy, tumor heterogeneity, and resistance mechanisms need to be addressed to improve therapeutic responsiveness. Future research should focus on elucidating the complex interplay between different PRMTs. Additionally, more selective inhibitors need to be developed, and PRMT-targeted therapies should be integrated with existing treatment modalities. By overcoming limitations in selectivity and toxicity, PRMT inhibitors could be a valuable addition to the therapeutic arsenal against breast cancer.

## CRediT authorship contribution statement

**Kamohelo Maphalala:** Writing – review & editing, Writing – original draft. **Dakalo Portia Ramali:** Writing – review & editing. **Lorraine Tshegofatso Maebele:** Writing – review & editing. **Thanyani Victor Mulaudzi:** Writing – review & editing. **Peace Mabeta:** Writing – review & editing. **Zodwa Dlamini:** Writing – review & editing. **Botle Precious Damane:** Writing – review & editing, Writing – original draft, Conceptualization.

## Informed consent

Not applicable.

## Ethical approval

Not applicable.

## Funding

This work was funded by the 10.13039/501100001321National Research Foundation [Grant number 107088], South African Medical Research Council (SAMRC) Researcher Development Award and the 10.13039/501100001343University of Pretoria Capacity Development Program under BP Damane.

## Declaration of competing interest

The authors declare the following financial interests/personal relationships which may be considered as potential competing interestsBotle Precious Damane reports financial support was provided by 10.13039/501100001321National Research Foundation. If there are other authors, they declare that they have no known competing financial interests or personal relationships that could have appeared to influence the work reported in this paper.

## Data Availability

No data was used for the research described in the article.

## References

[1] Lugano R., Ramachandran M., Dimberg A. (2020 May). Tumor angiogenesis: causes, consequences, challenges and opportunities. Cell. Mol. Life Sci..

[2] Saman H., Raza S.S., Uddin S., Rasul K. (2020 May 6). Inducing angiogenesis, a key step in cancer vascularization, and treatment approaches. Cancers (Basel).

[3] Castañeda-Gill J.M., Vishwanatha J.K. (2016). Antiangiogenic mechanisms and factors in breast cancer treatment. J. Carcinog..

[4] Folkman J., Browder T., Palmblad J. (2001 July). Angiogenesis research: guidelines for translation to clinical application. Thromb. Haemost..

[5] Tannock I.F. (1968 June). The relation between cell proliferation and the vascular system in a transplanted mouse mammary tumour. Br. J. Cancer.

[6] Hanahan D., Folkman J. (1996 August 9). Patterns and emerging mechanisms of the angiogenic switch during tumorigenesis. Cell.

[7] Shalaby F., Rossant J., Yamaguchi T.P. (1995 July 6). Failure of blood-island formation and vasculogenesis in Flk-1-deficient mice. Nature.

[8] Papetti M., Herman I.M. (2002 May). Mechanisms of normal and tumor-derived angiogenesis. Am. J. Physiol. Cell Physiol..

[9] Liekens S., De Clercq E., Neyts J. (2001 February 1). Angiogenesis: regulators and clinical applications. Biochem. Pharmacol..

[10] Ayoub N.M., Jaradat S.K., Al-Shami K.M., Alkhalifa A.E. (2022). Targeting angiogenesis in breast cancer: current evidence and future perspectives of novel anti-angiogenic approaches. Front. Pharmacol..

[11] Liu Z.-L., Chen H.-H., Zheng L.-L., Sun L.-P., Shi L. (2023 2023/05/11). Angiogenic signaling pathways and anti-angiogenic therapy for cancer. Signal Transduct. Targeted Ther..

[12] Subramaniam V.A., Yehya A.H.S., Cheng Wk (2019). Epigenetics: the master control of endothelial cell fate in cancer. Life Sci..

[13] Li Y., Qu X., Cao B. (2020 July). Selectively suppressing tumor angiogenesis for targeted breast cancer therapy by genetically engineered phage. Adv Mater.

[14] Sasich L.D., Sukkari S.R. (2012 October). The US FDAs withdrawal of the breast cancer indication for Avastin (bevacizumab). Saudi Pharm. J..

[15] Gacche R.N., Meshram R.J. (2014 August). Angiogenic factors as potential drug target: efficacy and limitations of anti-angiogenic therapy. Biochim. Biophys. Acta.

[16] de Heer E.C., Jalving M., Harris A.L. (2020 October 1). HIFs, angiogenesis, and metabolism: elusive enemies in breast cancer. J. Clin. Investig..

[17] Zou G., Zhang X., Wang L. (2020). Herb-sourced emodin inhibits angiogenesis of breast cancer by targeting VEGFA transcription. Theranostics.

[18] Li Y., Lin M., Wang S., Cao B., Li C., Li G. (2022). Novel angiogenic regulators and anti-angiogenesis drugs targeting angiogenesis signaling pathways: perspectives for targeting angiogenesis in lung cancer. Front. Oncol..

[19] Aspriţoiu V.M., Stoica I., Bleotu C., Diaconu C.C. (2021). Epigenetic regulation of angiogenesis in development and tumors progression: potential implications for cancer treatment. Front. Cell Dev. Biol..

[20] Dai W., Zhang J., Li S. (2022). Protein arginine methylation: an emerging modification in cancer immunity and immunotherapy. Front. Immunol..

[21] Ye Q., Zhang J., Zhang C. (2022 May 9). Endothelial PRMT5 plays a crucial role in angiogenesis after acute ischemic injury. JCI Insight.

[22] Morettin A., Baldwin R.M., Côté J. (2015). Arginine methyltransferases as novel therapeutic targets for breast cancer. Mutagenesis.

[23] Wang Y., Bedford M.T. (2023 April 26). Effectors and effects of arginine methylation. Biochem. Soc. Trans..

[24] Pham H.Q.H., Tao X., Yang Y. (2023). Protein arginine methylation in transcription and epigenetic regulation. Front Epigenet Epigenomics.

[25] Srour N., Khan S., Richard S. (2022). The influence of arginine methylation in immunity and inflammation. J. Inflamm. Res..

[26] Brobbey C., Liu L., Yin S., Gan W. (2022 August 29). The role of protein arginine methyltransferases in DNA damage response. Int. J. Mol. Sci..

[27] Poulard C., Corbo L., Le Romancer M. (2016 October 11). Protein arginine methylation/demethylation and cancer. Oncotarget.

[28] Ruben E., Paula L., Isabel R., Anica D. (2012). Methylation.

[29] Chang K., Gao D., Yan J., Lin L., Cui T., Lu S. (2023 October). Critical roles of protein arginine methylation in the central nervous system. Mol. Neurobiol..

[30] Zhang J., Jing L., Li M., He L., Guo Z. (2019 May). Regulation of histone arginine methylation/demethylation by methylase and demethylase. Mol. Med. Rep..

[31] Hernando C.E., Sanchez S.E., Mancini E., Yanovsky M.J. (2015 March 17). Genome wide comparative analysis of the effects of PRMT5 and PRMT4/CARM1 arginine methyltransferases on the Arabidopsis thaliana transcriptome. BMC Genom..

[32] Kim H., Ronai Z.A. (2020 July 13). PRMT5 function and targeting in cancer. Cell Stress.

[33] Samuel S.F., Barry A., Greenman J., Beltran-Alvarez P. (2021 April). Arginine methylation: the promise of a “silver bullet” for brain tumours?. Amino Acids.

[34] Thiebaut C., Eve L., Poulard C., Le Romancer M. (2021 October 27). Structure, activity, and function of PRMT1. Life.

[35] Goulet I., Gauvin G., Boisvenue S., Côté J. (2007 November 9). Alternative splicing yields protein arginine methyltransferase 1 isoforms with distinct activity, substrate specificity, and subcellular localization. J. Biol. Chem..

[36] Baldwin R.M., Morettin A., Côté J. (2014 May 26). Role of PRMTs in cancer: could minor isoforms be leaving a mark?. World J. Biol. Chem..

[37] Hwang J.W., Cho Y., Bae G.-U., Kim S.-N., Kim Y.K. (2021 May). Protein arginine methyltransferases: promising targets for cancer therapy. Exp. Mol. Med..

[38] Bradley R.K., Anczuków O. (2023 March). RNA splicing dysregulation and the hallmarks of cancer. Nat. Rev. Cancer.

[39] Hwang J.W., Kim S.-N., Myung N. (2020 2020/08/05). PRMT5 promotes DNA repair through methylation of 53BP1 and is regulated by Src-mediated phosphorylation. Commun. Biol..

[40] Lattouf H., Kassem L., Jacquemetton J. (2019). LKB1 regulates PRMT5 activity in breast cancer. Int. J. Cancer.

[41] Zhang H.-T., Zeng L.-F., He Q.-Y., Tao W.A., Zha Z.-G., Hu C.-D. (2016 2016/02/01). The E3 ubiquitin ligase CHIP mediates ubiquitination and proteasomal degradation of PRMT5. Biochim. Biophys. Acta Mol. Cell Res..

[42] Bhuripanyo K., Wang Y., Liu X. (2018 January). Identifying the substrate proteins of U-box E3s E4B and CHIP by orthogonal ubiquitin transfer. Sci. Adv..

[43] Li X., Song Y., Mu W., Hou X., Ba T., Ji S. (2024). Dysregulation of arginine methylation in tumorigenesis. Front. Mol. Biosci..

[44] Zhu Y., Xia T., Chen D.-Q. (2024). Promising role of protein arginine methyltransferases in overcoming anti-cancer drug resistance. Drug Resist. Updates.

[45] Zhao Y., Lu Q., Li C. (2019 2019/05/01). PRMT1 regulates the tumour-initiating properties of esophageal squamous cell carcinoma through histone H4 arginine methylation coupled with transcriptional activation. Cell Death Dis..

[46] Price O.M., Thakur A., Ortolano A. (2021 November). Naturally occurring cancer-associated mutations disrupt oligomerization and activity of protein arginine methyltransferase 1 (PRMT1). J. Biol. Chem..

[47] Smith E., Zhou W., Shindiapina P., Sif S., Li C., Baiocchi R.A. (2018 June). Recent advances in targeting protein arginine methyltransferase enzymes in cancer therapy. Expert Opin. Ther. Targets.

[48] Wei H., Mundade R., Lange K.C., Lu T. (2014). Protein arginine methylation of non-histone proteins and its role in diseases. Cell Cycle.

[49] Rasheed S., Bouley R.A., Yoder R.J., Petreaca R.C. (2023 March 23). Protein arginine methyltransferase 5 (PRMT5) mutations in cancer cells. Int. J. Mol. Sci..

[50] Wu K., Niu C., Liu H., Fu L. (2023 May). Research progress on PRMTs involved in epigenetic modification and tumour signalling pathway regulation. Int. J. Oncol..

[51] Chen Z., Gan J., Wei Z. (2022). The emerging role of PRMT6 in cancer. Front. Oncol..

[52] Baeriswyl V., Christofori G. (2009 October). The angiogenic switch in carcinogenesis. Semin. Cancer Biol..

[53] Madu C.O., Wang S., Madu C.O., Lu Y. (2020). Angiogenesis in breast cancer progression, diagnosis, and treatment. J. Cancer.

[54] Cheng H.-W., Chen Y.-F., Wong J.-M. (2017 February 7). Cancer cells increase endothelial cell tube formation and survival by activating the PI3K/Akt signalling pathway. J. Exp. Clin. Cancer Res..

[55] Hartsough E., Shelke R.R.J., Amraei R., Aryan Z., Lotfollahzadeh S., Rahimi N. (2022 August 19). PRMT4-mediated arginine methylation promotes tyrosine phosphorylation of VEGFR-2 and regulates filopodia protrusions. iScience.

[56] Herbert S.P., Odell A.F., Ponnambalam S., Walker J.H. (2009 February 27). Activation of cytosolic phospholipase A2-{alpha} as a novel mechanism regulating endothelial cell cycle progression and angiogenesis. J. Biol. Chem..

[57] Kamesaki H., Nishizawa K., Michaud G.Y., Cossman J., Kiyono T. (1998). TGF-β1 induces the cyclin-dependent kinase inhibitor p27Kip1 mRNA and protein in murine B cells. J. Immunol..

[58] Liu A., Yu C., Qiu C. (2023 2023/05/01). PRMT5 methylating SMAD4 activates TGF-β signaling and promotes colorectal cancer metastasis. Oncogene.

[59] Ding M.-J., Su K.E., Cui G.-Z. (2016 2016/05/09). Association between transforming growth factor-β1 expression and the clinical features of triple negative breast cancer. Oncol. Lett..

[60] Grau A.M., Wen W., Ramroopsingh D.S. (2008 November). Circulating transforming growth factor-beta-1 and breast cancer prognosis: results from the Shanghai breast cancer study. Breast Cancer Res. Treat..

[61] Wei H., Liu Y., Min J. (2019 November). Protein arginine methyltransferase 1 promotes epithelial-mesenchymal transition via TGF-β1/Smad pathway in hepatic carcinoma cells. Neoplasma.

[62] Mijit M., Caracciolo V., Melillo A., Amicarelli F., Giordano A. (2020 March 8). Role of p53 in the regulation of cellular senescence. Biomolecules.

[63] Varna M., Bousquet G., Plassa L.-F., Bertheau P., Janin A. (2011). TP53 status and response to treatment in breast cancers. J. Biomed. Biotechnol..

[64] Liu L.-M., Tang Q., Hu X. (2021 August 5). Arginine methyltransferase PRMT1 regulates p53 activity in breast cancer. Life.

[65] Neault M., Mallette F.A., Vogel G., Michaud-Levesque J., Richard S. (2012 October). Ablation of PRMT6 reveals a role as a negative transcriptional regulator of the p53 tumor suppressor. Nucleic Acids Res..

[66] Scoumanne A., Zhang J., Chen X. (2009). PRMT5 is required for cell-cycle progression and p53 tumor suppressor function. Nucleic Acids Res..

[67] Wang S., Li X., Parra M., Verdin E., Bassel-Duby R., Olson E.N. (2008 June 3). Control of endothelial cell proliferation and migration by VEGF signaling to histone deacetylase 7. Proc. Natl. Acad. Sci. U. S. A..

[68] Martinez S., Sentis S., Poulard C., Trédan O., Le Romancer M. (2024 August 14). Role of PRMT1 and PRMT5 in breast cancer. Int. J. Mol. Sci..

[69] Lamalice L., Le Boeuf F., Huot J. (2007 March 30). Endothelial cell migration during angiogenesis. Circ. Res..

[70] Michaelis U.R. (2014 November). Mechanisms of endothelial cell migration. Cell. Mol. Life Sci..

[71] Rundhaug J.E. (2005 April-June). Matrix metalloproteinases and angiogenesis. J. Cell Mol. Med..

[72] Baldwin R.M., Haghandish N., Daneshmand M. (2015 February 20). Protein arginine methyltransferase 7 promotes breast cancer cell invasion through the induction of MMP9 expression. Oncotarget.

[73] Kim N.H., Kim S.-N., Seo D.-W., Han J.-W., Kim Y.K. (2013 2013/03/01). PRMT6 overexpression upregulates TSP-1 and downregulates MMPs: its implication in motility and invasion. Biochem. Biophys. Res. Commun..

[74] Karar J., Maity A. (2011). PI3K/AKT/mTOR pathway in angiogenesis. Front. Mol. Neurosci..

[75] Im E., Kazlauskas A. (2006). Regulating angiogenesis at the level of PtdIns-4,5-P2. EMBO J..

[76] Tan L., Xiao K., Ye Y. (2020 May 11). High PRMT5 expression is associated with poor overall survival and tumor progression in bladder cancer. Aging (Albany, New York).

[77] Al Kawas H., Saaid I., Jank P. (2022 April). How VEGF-A and its splice variants affect breast cancer development—clinical implications. Cell Oncol (Dordr).

[78] Brogowska K.K., Zajkowska M., Mroczko B. (2023). Vascular endothelial growth factor ligands and receptors in breast cancer. J. Clin. Med..

[79] Yang Y., Cao Y. (2022 November). The impact of VEGF on cancer metastasis and systemic disease. Semin. Cancer Biol..

[80] Srabovic N., Mujagic Z., Mujanovic-Mustedanagic J. (2013). Vascular endothelial growth factor receptor-1 expression in breast cancer and its correlation to vascular endothelial growth factor A. Int. J. Breast Cancer.

[81] Almumen M. (2015 01/01). Immunohistochemical expression of VEGF in relation to other pathological parameters of breast carcinoma. J. Cancer Ther..

[82] Wang X., Bove A.M., Simone G., Ma B. (2020). Molecular bases of VEGFR-2-Mediated physiological function and pathological role. Front. Cell Dev. Biol..

[83] Lucia N., Maria A., Federico B., Dan S., Agneta S. (2017). Physiologic and Pathologic Angiogenesis.

[84] Wu H.M., Yuan Y., Zawieja D.C., Tinsley J., Granger H.J. (1999 February). Role of phospholipase C, protein kinase C, and calcium in VEGF-induced venular hyperpermeability. Am. J. Physiol..

[85] Björndahl M., Cao R., Eriksson A., Cao Y. (2004). Blockage of VEGF-induced angiogenesis by preventing VEGF secretion. Circ. Res..

[86] Zhang X., Wang K., Feng X. (2021 2021/11/09). PRMT3 promotes tumorigenesis by methylating and stabilizing HIF1α in colorectal cancer. Cell Death Dis..

[87] Harrison M.J., Tang Y.H., Dowhan D.H. (2010 April). Protein arginine methyltransferase 6 regulates multiple aspects of gene expression. Nucleic Acids Res..

[88] Mann M., Zou Y., Chen Y., Brann D., Vadlamudi R. (2014 March). PELP1 oncogenic functions involve alternative splicing via PRMT6. Mol. Oncol..

[89] Avasarala S., Wu P.-Y., Khan S.Q. (2020 January). PRMT6 promotes lung tumor progression via the alternate activation of tumor-associated macrophages. Mol. Cancer Res..

[90] Li L., Zhang Z., Ma T., Huo R. (2016 July). PRMT1 regulates tumor growth and metastasis of human melanoma via targeting ALCAM. Mol. Med. Rep..

[91] Zhou G., Zhang C., Peng H. (2024 January 10). PRMT3 methylates HIF-1alpha to enhance the vascular calcification induced by chronic kidney disease. Mol Med.

[92] Zheng Y., Ji H., Yi W. (2023 July 3). PRMT5 facilitates angiogenesis and EMT via HIF-1alpha/VEGFR/Akt signaling axis in lung cancer. Aging (Albany, New York).

[93] Touat M., Ileana E., Postel-Vinay S., André F., Soria J.-C. (2015 June 15). Targeting FGFR signaling in cancer. Clin. Cancer Res..

[94] Santolla M.F., Maggiolini M. (2020 October 18). The FGF/FGFR system in breast cancer: oncogenic features and therapeutic perspectives. Cancers (Basel).

[95] Tenhagen M., van Diest P.J., Ivanova I.A., van der Wall E., van der Groep P. (2012 August). Fibroblast growth factor receptors in breast cancer: expression, downstream effects, and possible drug targets. Endocr. Relat. Cancer.

[96] Jing P., Zhao N., Ye M. (2018 July 28). Protein arginine methyltransferase 5 promotes lung cancer metastasis via the epigenetic regulation of miR-99 family/FGFR3 signaling. Cancer Lett..

[97] Wang Q., Xu J., Li Y. (2018). Identification of a novel protein arginine methyltransferase 5 inhibitor in non-small cell lung cancer by structure-based virtual screening. Front. Pharmacol..

[98] Zhang B., Dong S., Zhu R. (2015 September 8). Targeting protein arginine methyltransferase 5 inhibits colorectal cancer growth by decreasing arginine methylation of eIF4E and FGFR3. Oncotarget.

[99] Sheng X., Wang Z. (2016 2016/08/02). Protein arginine methyltransferase 5 regulates multiple signaling pathways to promote lung cancer cell proliferation. BMC Cancer.

[100] Motolani A., Martin M., Sun M., Lu T. (2021 October 12). The structure and functions of PRMT5 in Human Diseases. Life.

[101] Davis L., Recktenwald M., Hutt E. (2022 February 28). Targeting HIF-2α in the tumor microenvironment: redefining the role of HIF-2α for solid cancer therapy. Cancers (Basel).

[102] Emami Nejad A., Najafgholian S., Rostami A. (2021 2021/01/20). The role of hypoxia in the tumor microenvironment and development of cancer stem cell: a novel approach to developing treatment. Cancer Cell Int..

[103] Al Tameemi W., Dale T.P., Al-Jumaily R.M.K., Forsyth N.R. (2019). Hypoxia-modified cancer cell metabolism. Front. Cell Dev. Biol..

[104] Bui B.P., Nguyen P.L., Lee K., Cho J. (2022). Hypoxia-inducible factor-1: a novel therapeutic target for the management of cancer, drug resistance, and cancer-related pain. Cancers.

[105] Albanese A., Daly L.A., Mennerich D., Kietzmann T., Sée V. (2020 December 29). The role of hypoxia-inducible factor post-translational modifications in regulating its localisation, stability, and activity. Int. J. Mol. Sci..

[106] Masoud G.N., Li W. (2015 September). HIF-1α pathway: role, regulation and intervention for cancer therapy. Acta Pharm. Sin. B.

[107] Liao Y., Luo Z., Lin Y. (2022 2022/11/09). PRMT3 drives glioblastoma progression by enhancing HIF1A and glycolytic metabolism. Cell Death Dis..

[108] Dong F., Sun X., Su J. (2024). Hypoxia-inducible PRMT2 addiction in glioblastomas. Cell. Signal..

[109] Lafleur V.N., Richard S., Richard D.E. (2014 March). Transcriptional repression of hypoxia-inducible factor-1 (HIF-1) by the protein arginine methyltransferase PRMT1. Mol. Biol. Cell.

[110] Hü Kaniskan, Martini M.L., Jin J. (2018 February 14). Inhibitors of protein methyltransferases and demethylases. Chem Rev.

[111] Wang S.M., Dowhan D.H., Muscat G.E.O. (2019 April 1). Epigenetic arginine methylation in breast cancer: emerging therapeutic strategies. J. Mol. Endocrinol..

[112] Gerhart S.V., Kellner W.A., Thompson C. (2018 June 26). Activation of the p53-MDM4 regulatory axis defines the anti-tumour response to PRMT5 inhibition through its role in regulating cellular splicing. Sci. Rep..

[113] Siu L.L., Rasco D.W., Vinay S.P. (2019).

[114] Vinet M., Suresh S., Maire V. (2019 May). Protein arginine methyltransferase 5: a novel therapeutic target for triple-negative breast cancers. Cancer Med..

[115] Elkin M., Orgel A., Kleinman H.K. (2004 June 2). An angiogenic switch in breast cancer involves estrogen and soluble vascular endothelial growth factor receptor 1. J. Natl. Cancer Inst..

[116] Qi C., Chang J., Zhu Y., Yeldandi A.V., Rao S.M., Zhu Y.-J. (2002 August 9). Identification of protein arginine methyltransferase 2 as a coactivator for estrogen receptor alpha. J. Biol. Chem..

[117] Shen Y., Zhong J., Liu J. (2018 June). Protein arginine N-methyltransferase 2 reverses tamoxifen resistance in breast cancer cells through suppression of ER-α36. Oncol. Rep..

[118] Sun Y., Chung H.H., Woo A.R.E., Lin V.C.-L. (2014 2014/09/01). Protein arginine methyltransferase 6 enhances ligand-dependent and -independent activity of estrogen receptor α via distinct mechanisms. Biochim. Biophys. Acta Mol. Cell Res..

[119] Le Romancer M., Treilleux I., Leconte N. (2008). Regulation of estrogen rapid signaling through arginine methylation by PRMT1. Mol Cell.

[120] Mei S., Ge S., Wang J. (2021). PRMT5 promotes progression of endometrioid adenocarcinoma via ERα and cell cycle signaling pathways. J Pathol Clin Res.

[121] Clemons G.A., Silva A.C.E., Acosta C.H. (2024). Protein arginine methyltransferase 4 modulates nitric oxide synthase uncoupling and cerebral blood flow in Alzheimer's disease. J. Cell. Physiol..

[122] Tupurani Mohini A., Padala C., Hanumanth Surekha R., Seyed Soheil Saeedi S. (2017). Nitric Oxide Synthase.

[123] Couto E.Silva A., Wu C.Y.-C., Citadin C.T. (2020 March). Protein arginine methyltransferases in cardiovascular and neuronal function. Mol. Neurobiol..

[124] Choudhari S.K., Chaudhary M., Bagde S., Gadbail A.R., Joshi V. (2013 May 30). Nitric oxide and cancer: a review. World J. Surg. Oncol..

[125] Goveia J., Stapor P., Carmeliet P. (2014 September). Principles of targeting endothelial cell metabolism to treat angiogenesis and endothelial cell dysfunction in disease. EMBO Mol. Med..

[126] Watts J., Minden M.D., Bachiashvili K. (2024). Phase I/II study of the clinical activity and safety of GSK3326595 in patients with myeloid neoplasms. Ther Adv Hematol.

[127] El-Khoueiry A.B., Clarke J., Neff T. (2023 August). Phase 1 study of GSK3368715, a type I PRMT inhibitor, in patients with advanced solid tumors. Br. J. Cancer.

[128] Fedoriw A., Rajapurkar S.R., O'Brien S. (2019 July 8). Anti-tumor activity of the Type I PRMT inhibitor, GSK3368715, synergizes with PRMT5 inhibition through MTAP loss. Cancer Cell.

[129] Chen Y., Shao X., Zhao X. (2021 December). Targeting protein arginine methyltransferase 5 in cancers: roles, inhibitors and mechanisms. Biomed. Pharmacother..

[130] Katsuno Y., Qin J., Oses-Prieto J. (2018 August 24). Arginine methylation of SMAD7 by PRMT1 in TGF-beta-induced epithelial-mesenchymal transition and epithelial stem-cell generation. J. Biol. Chem..

[131] Qin J., Xu J. (2022 December). Arginine methylation in the epithelial-to-mesenchymal transition. FEBS J..

[132] Bhandari K., Ding W.-Q. (2024 April 2). Protein arginine methyltransferases in pancreatic ductal adenocarcinoma: new molecular targets for therapy. Int. J. Mol. Sci..

[133] Dakroub R., Huard S., Hajj-Younes Y. (2023). Therapeutic advantage of targeting PRMT5 in combination with chemotherapies or EGFR/HER2 inhibitors in triple-negative breast cancers. Breast Cancer.

[134] Wang Z., Kong J., Wu Y. (2018 April). PRMT5 determines the sensitivity to chemotherapeutics by governing stemness in breast cancer. Breast Cancer Res. Treat..

[135] Carter J., Hulse M., Sivakumar M. (2023 November 6). PRMT5 inhibitors regulate DNA damage repair pathways in cancer cells and improve response to PARP inhibition and chemotherapies. Cancer Res. Commun..

[136] Brehmer D., Beke L., Wu T. (2021 December). Discovery and pharmacological characterization of JNJ-64619178, a novel small-molecule inhibitor of PRMT5 with potent antitumor activity. Mol. Cancer Therapeut..

[137] Vieito M., Moreno V., Spreafico A. (2023 September 15). Phase 1 study of JNJ-64619178, a protein arginine methyltransferase 5 inhibitor, in advanced solid tumors. Clin. Cancer Res..

[138] Trnkova L., Buocikova V., Mego M. (2024). Epigenetic deregulation in breast cancer microenvironment: implications for tumor progression and therapeutic strategies. Biomed. Pharmacother..

[139] Hu H., Qian K., Ho M.-C., Zheng Y.G. (2016). Small molecule inhibitors of protein arginine methyltransferases. Expet Opin. Invest. Drugs.

[140] Tong C., Chang X., Qu F. (2024). Overview of the development of protein arginine methyltransferase modulators: achievements and future directions. Eur. J. Med. Chem..

[141] Fulton M.D., Brown T., Zheng Y.G. (2018 December). Mechanisms and inhibitors of histone arginine methylation. Chem. Rec..

